# PLAG‐Family Amplified CNS Embryonal Tumour With PLAG1 Immunohistochemical Expression: Expanding the Spectrum of Diagnostic Tools

**DOI:** 10.1111/nan.70017

**Published:** 2025-04-10

**Authors:** Antonio d'Amati, Flavia Adotti, Francesca Gianno, Domenico Cicala, Eugenio Covelli, Giuseppe Cinalli, Vittoria D'Onofrio, Maria Elena Errico, Lucia Quaglietta, Sabina Barresi, Sabrina Rossi, Evelina Miele, Manila Antonelli

**Affiliations:** ^1^ Unit of Human Anatomy and Histology, Department of Translational Biomedicine and Neuroscience (DiBraiN) University of Bari “Aldo Moro” Bari Italy; ^2^ Unit of Anatomical Pathology, Department of Radiology, Oncology and Anatomical Pathology University La Sapienza Rome Italy; ^3^ Anatomical Pathology Unit Fondazione Policlinico Universitario "A. Gemelli" IRCCS, Università Cattolica S. Cuore Rome Italy; ^4^ Network Oncology and Precision Medicine, Department of Experimental Medicine University La Sapienza Rome Italy; ^5^ IRCCS Neuromed Pozzilli Italy; ^6^ Pediatric Neuroradiology Unit Santobono‐Pausilipon Children's Hospital, AORN Naples Italy; ^7^ Pediatric Neurosurgery Unit Santobono‐Pausilipon Children's Hospital, AORN Naples Italy; ^8^ Department of Pathology AORN Santobono‐Pausilipon, Pediatric Hospital Naples Italy; ^9^ Department of Pediatric Oncology Santobono‐Pausilipon Pediatric Hospital Naples Italy; ^10^ Pathology Unit, Department of Laboratories Bambino Gesù Children's Hospital, IRCCS Rome Italy; ^11^ Department of Pediatric Onco‐Hematology and Cell and Gene Therapy Bambino Gesù Children's Hospital, IRCCS Rome Italy

**Keywords:** CNS embryonal tumours, CNS tumours, PLAG(L)‐altered CNS tumours, PLAG1, PLAGL1, PLAGL2

PLAG(L)‐altered tumours have recently emerged as a novel category of Central Nervous System (CNS) tumours, although they are not yet included in the most recent WHO Classification. In 2022, genome‐wide DNA‐methylation analysis of cases previously diagnosed as CNS embryonal tumours, high‐grade gliomas, or unclassifiable neoplasms led to the identification of the ‘CNS embryonal tumour with PLAG‐family amplification’ methylation class, characterised by *PLAGL1* or *PLAGL2* amplification [1]. In 2023, two embryonal tumours harbouring *PLAG1* fusions, which share clinical, radiological, histopathological, immunohistochemical and epigenetic features with CNS embryonal tumours with PLAG‐family amplification, were also reported [2]. Here, we describe a case of CNS embryonal tumour with *PLAGL2* amplification, contributing new evidence to the characterisation of this evolving entity. To the best of our knowledge, we also present the first report of PLAG1 immunostaining positivity, suggesting its potential as a useful diagnostic tool in routine practice.

The case involves a 3‐year‐old boy with a tumour located in the third ventricle. MRI revealed a large, heterogeneous mass, with cystic areas and strong contrast enhancement, leading to obstructive hydrocephalus (Figure 1). Histopathological examination showed a primitive embryonal‐like proliferation, consisting of sheets of monotonous small cells with round or oval hyperchromatic nuclei and scant pale cytoplasm (Figure 2A,B). Scattered pleomorphic elements were also present, characterised by large eccentric hyperchromatic nuclei and abundant eosinophilic cytoplasm (Figure 2C). No haemorrhage, microcystic change, rosettes, pseudorosettes or rhabdoid components were observed. Necrosis, microvascular proliferation and a high mitotic count were noted. Immunohistochemistry revealed positivity for synaptophysin (Figure 2E), patchy GFAP expression and negativity for OLIG2 (Figure 2D). INI1 and BRG1 expression were preserved, while LIN28A and BCOR were negative. EMA showed focal positivity with a ‘dot‐like’ pattern. Desmin was diffusely expressed in neoplastic cells, while other myogenic markers (SMA, caldesmon and calponin) were completely negative. PLAG1 immunostaining was also performed (1:100, clone 3B7, Novus Biologicals, Centennials, CO, USA), revealing nuclear positivity in more than 50% of tumour cells (Figure 2F). DNA‐methylation profiling (Heidelberg classifier, v12.5) classified the case as a CNS embryonal tumour with PLAG‐family amplification, with a calibrated match score of 0.99. Copy number variation (CNV) analysis from the EPIC array revealed a gain of chromosome 19 and amplification/rearrangement of chromosome 20 in a region encompassing the *PLAGL2* gene (Figure S1). RNA‐seq analysis did not identify any definitively pathogenic fusions but, consistent with previous findings, revealed multiple translocations and inversions involving chromosome 20 (q11.21–q11.23). These structural alterations may have generated fusion transcripts involving the contiguous *KIF3B* and *PLAGL2* genes—both located on chromosome 20q11.21—along with other partner genes. However, these transcripts were either out‐of‐frame or encoded putative chimeric proteins lacking essential domains, with no apparent biological function. Additionally, the amplification of the *PLAGL2* gene might be associated with the structural rearrangements affecting this region.

**FIGURE 1:**
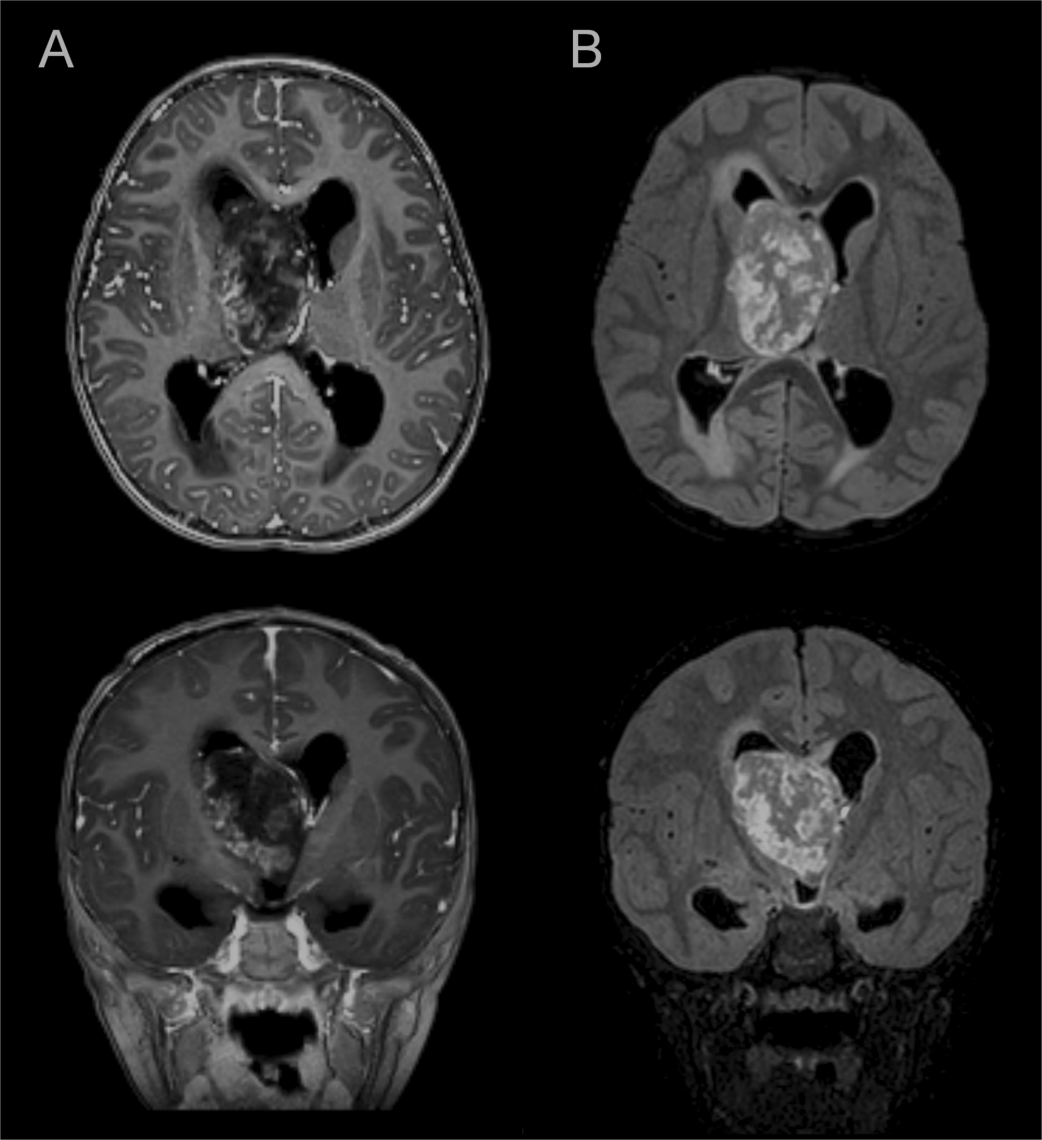
MR study. Contrast‐enhanced T1 weighted (A) and FLAIR (B) images on axial and coronal planes show a large tumour with endoventricular growth, close to the right caudothalamic groove; it shows heterogeneous structure with areas of hyperintense signal in FLAIR and patchy contrast enhancement. The lesion causes severe hydrocephalus due to obstruction of the foramina of Monro, with signs of intracranial hypertension and transependymal oedema.

**FIGURE 2:**
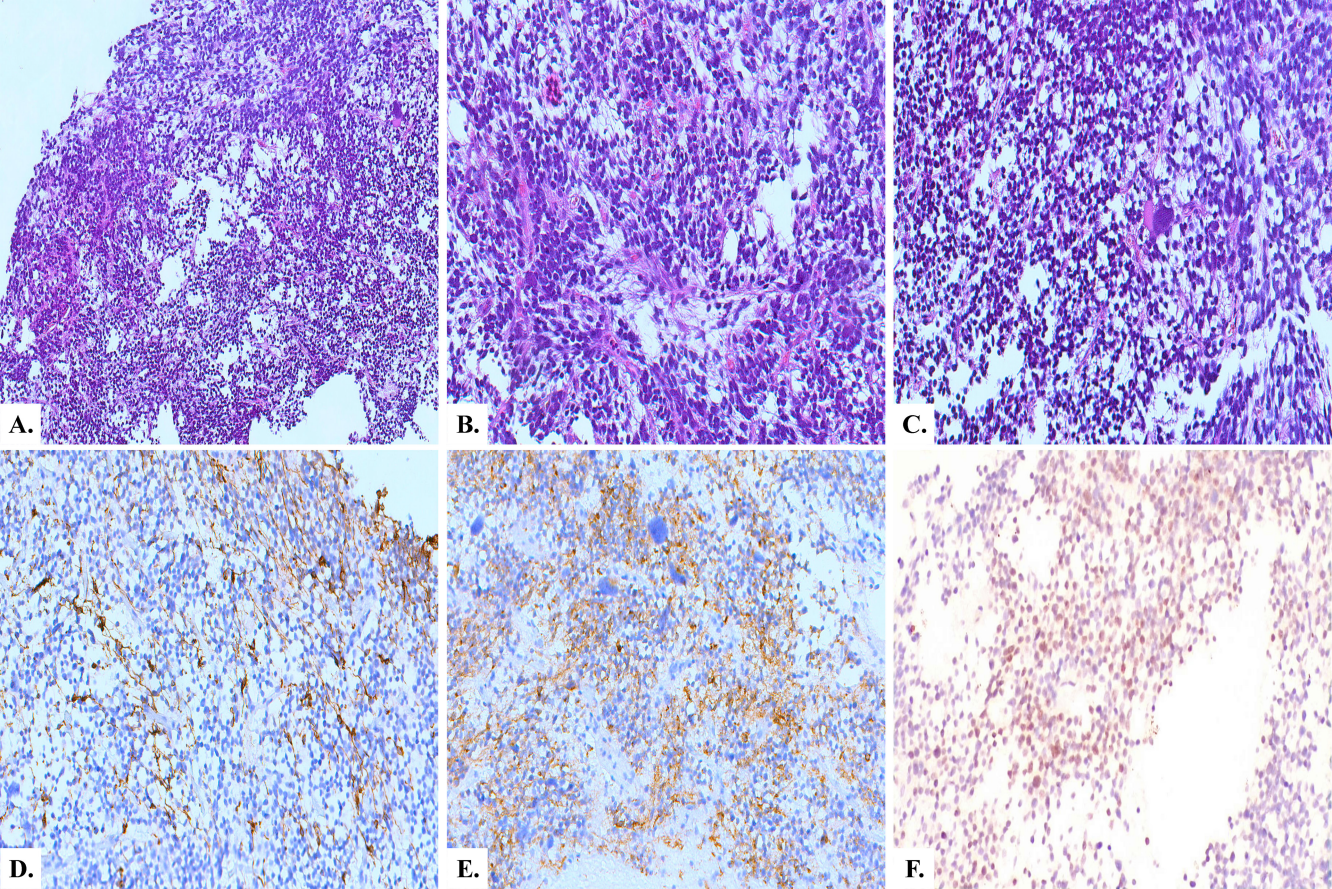
(A) Sheets of primitive embryonal‐like cells (HE, 100x). (B) Tumour cells with round/oval hyperchromatic nuclei and scarce pale cytoplasm (HE, 200x). (C) Scattered elements with enlarged hyperchromatic nuclei (HE, 200x). (D) Patchy GFAP expression (200x). (E) Diffuse granular synaptophysin positivity (200x). (F) PLAG1 nuclear expression in the majority of tumour cells (400x).

To date, PLAG‐family gene alterations have been implicated in two distinct CNS tumour types. *PLAGL1/2* amplification [1] and *PLAG1* fusions [2] have been reported in CNS embryonal tumours with PLAG‐family amplification, while *PLAGL1* fusions have been identified in neuroepithelial tumours with ependymoma‐like features [3, 4]. CNS tumours with *PLAGL1/2* amplification are primitive embryonal‐like proliferations with intermediate survival, affecting various CNS regions and typically lacking diffuse expression of glial or neuronal markers. The age of onset appears to correlate with the underlying molecular alteration: tumours with *PLAGL1* amplification are more commonly diagnosed in adolescents, whereas *PLAGL2*‐amplified tumours predominantly affect infants and toddlers [1]. The recently described CNS embryonal tumours with *PLAG1*‐fusion include only two hemispheric cases, one in a paediatric and one in an adult patient, exhibiting embryonal‐like morphology, focal epithelioid appearance and expression of neuronal differentiation markers [2]. Our case aligns with the epidemiological features reported by Keck et al. for *PLAGL2‐*amplified CNS embryonal tumours, as it occurred in a male infant and involved a supratentorial location. Immunohistochemically, the phenotype is also consistent with the literature; *PLAGL1/2*‐amplified CNS tumours are generally negative for GFAP and OLIG2, with only some *PLAGL2*‐amplified cases partially expressing glial markers, as well as EMA showing a dot‐like pattern. As previously described, we observed positivity for neuronal (synaptophysin) and myogenic (desmin) markers. More interestingly, we also noted aberrant expression of PLAG1 protein in the nuclei of most tumour cells, despite the case being classified as *PLAGL2‐*amplified CNS embryonal tumour, based on DNA‐methylation and CNV results. To our knowledge, PLAG1 immunostaining has not been previously investigated in CNS embryonal tumours with *PLAGL1/2*‐amplification. Given that *PLAG*‐family genes encode zinc finger proteins with homologous sequences, we assessed the potential for immunohistochemical cross‐reactivity by comparing the amino acid sequence of the PLAG1 epitope recognised by the antibody (clone 3B7, Novus Biologicals, Centennials, CO, USA) with the zinc finger domains of PLAGL2. Using the NIH BLAST protein database (https://blast.ncbi.nlm.nih.gov/Blast.cgi? PAGE = Proteins), we found 72% identity in the C2H2‐type 1 region (amino acid positions 68–92). This result supports our hypothesis that cross‐reactivity may explain the observed immunostaining and suggests that the PLAG1 antibody could be a valuable diagnostic tool for CNS embryonal tumours with *PLAG*‐family amplification. To evaluate the specificity of the PLAG1 antibody, we tested it on a tissue microarray (TMA) containing 20 CNS embryonal tumours, including 12 medulloblastomas, four atypical teratoid/rhabdoid tumours (AT/RT), two *FOXR2*‐activated neuroblastomas and two CNS embryonal tumours with *BCOR*‐ITD mutations. A pleomorphic adenoma of the parotid gland was used as a positive control for PLAG1. In all cases of CNS embryonal tumours analysed, the antibody showed no reactivity, resulting in a completely negative staining profile across all tumour types. Specifically, in every case of the TMA, there was a complete absence of any positive staining pattern, with no detectable signal or any sign of reactivity to the PLAG1 antibody in the tumour cells, further confirming the specificity of the antibody in this context (Figure S2). However, additional studies are required to validate its sensitivity and specificity more thoroughly, as well as to explore its expression in other types of CNS tumours. Furthermore, it would be interesting to assess PLAG1 expression in embryonal tumours with *PLAG1* fusions, such as the two cases described by Tauziede‐Espariat et al. [2], and to compare these with *PLAGL1/2‐*amplified tumours.

In conclusion, we report, for the first time, a CNS embryonal tumour with PLAG1 immunohistochemical expression, sharing clinical, radiological, histopathological, immunohistochemical and epigenetic features with CNS embryonal tumours with *PLAG*‐family amplification. If validated in future studies, the PLAG1 antibody could expand the diagnostic toolkit for this recently described entity, which requires further confirmation through molecular analyses.

## Author Contributions

Antonio d'Amati and Manila Antonelli: conception and design of the work. Antonio d'Amati, Flavia Adotti, Francesca Gianno, Domenico Cicala, Eugenio Covelli, Giuseppe Cinalli, Vittoria D'Onofrio, Maria Elena Errico, Lucia Quaglietta, Sabina Barresi, Sabrina Rossi and Evelina Miele: acquisition, analysis and interpretation of data. Flavia Adotti, Antonio d'Amati: drafting of the manuscript. Antonio d'Amati and Manila Antonelli: revision of the manuscript. All authors read and approved the final manuscript.

## Ethics Statement

The study of the present case report has been performed in accordance with the Declaration of Helsinki.

## Consent

The patient's parents signed informed consent for diagnostic, therapeutic and research purposes, including the publication of any images or data included in this article.

## Conflicts of Interest

The authors declare no conflicts of interest.

## Supporting information


**Data S1** Supporting Information.

**Figure S1** CNV profiling detected the gain of Chromosome 19 and the rearrangement in Chromosome 20, presumably responsible for PLAGL2 amplification.

**Figure S2** PLAG1 staining specificity assessment. **A.** CNS embryonal tumours TMA (2x) shows no immunoreactivity. **B.** Medulloblastoma tissue core focus (200x) also demonstrates the complete absence of immunoreactivity. **C.** A section of pleomorphic adenoma of the parotid gland (positive control) shows a positive staining pattern for PLAG1 (200x).

## Data Availability

The data analysed during the current study are available from the corresponding author upon reasonable request.
