# Supplementary material for: PLAG‐Family Amplified CNS Embryonal Tumour With PLAG1 Immunohistochemical Expression: Expanding the Spectrum of Diagnostic Tools
Source: Neuropathol Appl Neurobiol. 2025 Apr 10;51(2):e70017. doi: 10.1111/nan.70017 (PMC11984066; doi:10.1111/nan.70017)
Supplement: Supplementary file 1 — Data S1 Supporting Information. [file NAN-51-e70017-s001.docx]

**Supporting Information**

**Supplementary Figure Legends**

**Figure S1.** CNV profiling detected the gain of Chromosome 19 and the rearrangement in Chromosome 20, presumably responsible for PLAGL2 amplification.

**Figure S2.** PLAG1 staining specificity assessment. **A.** CNS embryonal tumours TMA (2x) shows no immunoreactivity. **B.** Medulloblastoma tissue core focus (200x) also demonstrates the complete absence of immunoreactivity. **C.** A section of pleomorphic adenoma of the parotid gland (positive control) shows a positive staining pattern for PLAG1 (200x).
